# Reelin Protects against Colon Pathology via p53 and May Be a Biomarker for Colon Cancer Progression

**DOI:** 10.3390/biology11101406

**Published:** 2022-09-26

**Authors:** José M. Serrano-Morales, María D. Vázquez-Carretero, Pablo García-Miranda, Ana E. Carvajal, María L. Calonge, Anunciación A. Ilundain, María J. Peral

**Affiliations:** Departamento de Fisiología, Facultad de Farmacia, Universidad de Sevilla, 41012 Seville, Spain

**Keywords:** reelin, p53, Akt, colon cancer, colitis, DNMT-1, ApoER2

## Abstract

**Simple Summary:**

Colon cancer is a multifactorial disease involving genetic, environmental and lifestyle risk factors. Despite being one of the most common malignancies and a leading cause of cancer-related death worldwide, its underlying molecular mechanisms are scarcely known, highlighting the need to identify novel biomarkers for the clinical detection of its initiation and progression. The aim of the current study was to elucidate the role of the protein reelin in colon cancer initiation and progression using mouse models and human samples that extend from colitis or precancerous lesions to colon cancer. The results might contribute to understanding the mechanisms involved in colon cancer, and suggest that reelin may be a biomarker of colon pathology progression.

**Abstract:**

Previous observations made in human and mouse colons suggest that reelin protects the colon from pathology. In this study, we evaluated reelin expression during the transition from either colitis or precancerous lesions to colon cancer and tried to elucidate reelin regulation under these transition processes. Samples of healthy and pathological colons from humans and mice treated with either azoxymethane/dextran sulfate sodium (DSS) or azoxymethane alone were used. The relative abundances of reelin, DNMT-1 and ApoER2 mRNAs were determined by PCR in the colon samples cited above and in the tissue adjacent to mouse colon polyps and adenocarcinomas. In both, humans and mice, reelin mRNA abundance increased significantly in ulcerative colitis and slightly in polyps and decreased in adenomas and adenocarcinomas. Reelin expression was higher in the tissue adjacent to the colon adenocarcinoma and lower in the lesion itself. The reelin expression changes may result, at least in part, from those in DNMT-1 and appear to be independent of ApoER2. Lack of reelin downregulated p-Akt and p53 in healthy colon and prevented their increases in the inflamed colon, whereas it increased GSK-3β in DSS-untreated mice. In conclusion, reelin mRNA abundance depends on the severity of the colon pathology, and its upregulation in response to initial injuries might prevent the beginning of colon cancer, whereas reelin repression favors it. Increased p53 expression and activation may be involved in this protection. We also propose that changes in colon reelin abundance could be used to predict colon pathology progression.

## 1. Introduction

Colon cancer is a multifactorial disease involving genetic, environmental and lifestyle risk factors. The colon cancer that develops in people without genetic predisposition is named “sporadic colon cancer” and evolves through a complex multistep process, wherein the earliest neoplastic lesion, named aberrant crypt foci (ACF), can progress to polyps and, subsequently, adenomatous polyps or adenomas, then, finally, to cancer [1,2]. The colon cancer called “colitis-associated colon cancer” develops from chronic colon inflammation through an “ulcerative colitis-associated dysplasia–carcinoma sequence”; hence ulcerative colitis is considered a risk factor for suffering colon cancer [3,4]. Despite colon cancer being one of the most common malignancies diagnosed and a leading cause of cancer-related death worldwide [5], its underlying molecular mechanisms are scarcely known, highlighting the need to identify novel biomarkers for the clinical detection of colon initiation and progression.

Among the factors participating in inflammation and tumorigenesis are the tumor suppressor p53 transcription factor [6,7], the protein kinase B (PKB or Akt) and the glycogen synthase kinase-3β (GSK-3β) [8,9,10]. Accumulating observations indicate that p53 inhibits inflammation and prevents tumor initiation and progression [7,11]. In resting cells, the murine double minute 2 (MDM2) protein maintains p53 at low levels by inducing its degradation. Stressful events, such as inflammation, induce p53 Ser^15^ phosphorylation, which promotes its dissociation from MDM2 and, hence, increases its abundance by preventing its MDM2-dependent degradation [12,13]. The active form of GSK-3β activates MDM2 and produces p53 degradation, whereas activated Akt (phosphorylated at Ser^473^) inactivates GSK-3β. Therefore, Akt activation and subsequent GSK-3β inactivation inhibit p53 degradation, promoting its stabilization and accumulation [10].

Reelin is an extracellular matrix glycoprotein that was first known for its function in brain development as a controller of cortical neuron migration and positioning [14]. Several genetic and epigenetic mechanisms regulate reelin gene expression [15]. In neurons, it is reduced by both, the hypermethylation of its promoter and the overexpression of the methylating enzyme DNA methyltransferase 1 (DNMT-1), whereas it is increased by the knockdown of DNMT-1 [15]. Later studies reported reelin expression in a wide variety of nonneuronal tissues, mainly involved in organ development [16]. In addition to tissue homeostasis, reelin might have a role in diseases since altered expressions of reelin have been found in several pathologies, including cancer [16]. Reelin functions are mediated by its binding to specific receptors, which triggers the activation of downstream intracellular signals, including the activation of Akt [17,18].

We previously reported that reelin absence increases colitis severity and colon tumorigenesis in mice [19,20] and that reelin was upregulated in the colon of a mouse model of acute colitis and downregulated in human colon adenocarcinoma [19,21]. These reelin changes were the opposite of those in DNMT-1 expression and activity, which, via the hypomethylation of the reelin promoter in acute colitis and hypermethylation in colon adenocarcinoma, increases or represses reelin expression, respectively [19,21]. Apolipoprotein E receptor 2 (ApoER2 or LRP8) is a reelin receptor that downregulates reelin expression [22], and we found a negative correlation between the expression of this receptor and that of reelin in human colon adenocarcinoma [21].

Based on our observations, we suggested that reelin provides protection against intestinal pathology. We also hypothesized that the microenvironment accompanying the colon lesion could change the epithelial myofibroblasts from cells expressing high reelin (inflammation) [19], which protects against pathology, to those producing less or no reelin, which would allow for cancer development.

The aim of the current study was to elucidate the role of reelin in colon cancer initiation and progression. We also attempted to unravel the mechanism that regulates reelin expression during colon cancer progression, as well as the signaling molecules activated by reelin. For this purpose, a mouse model of sporadic and colitis-associated colon cancer was used. Human samples that correspond to the different stages of the adenoma–carcinoma sequence were also employed.

## 2. Materials and Methods

### 2.1. Human Tissue Samples

Embedded paraffin sections of colons from patients with ulcerative colitis, colon polyps (non-adenomatous polyps), colon adenomas (adenomatous polyps), colon adenocarcinomas (stage II/III sporadic colorectal cancer) and healthy colon located away from the adenocarcinoma region, were obtained from 40 patients (49–74-years-old), eight per each condition, who had undergone colon resection. The samples were provided by the “Biobanco del Sistema Sanitario Público de Andalucía, Hospital Universitario Virgen del Rocío, Sevilla, Spain”. Tissues were subjected to a pathological examination at the hospital to confirm the diagnosis of the pathology. The study was approved by the Ethic Committees of Sevilla University and Virgen del Rocío Hospital. Informed consent was obtained from all subjects involved in the study. The participants received adequate information related to the study from the hospital and signed the consent form.

### 2.2. Animals

C57BL6/J mice aged 2–3 months and B6C3Fe wild-type and reeler (rl−/rl−) mice aged 3 months were used. Heterozygous (rl+/rl−) mice were purchased from Jackson Laboratories (Bar Harbor, ME) through Charles River Laboratories, Spain. Wild-type (rl+/rl+) and homozygous reeler (rl−/rl−) mice were obtained via heterozygous crossings. Animals were housed in a 12:12 light–dark cycle and fed ad libitum with either a Global rodent diet for C57BL6/J mice or Global 2019-extruded rodent diet for B6C3Fe mice (Harlan Iberica S.L.), with free access to water. Reeler mice were genotyped by PCR analysis of genomic DNA, as described in [23]. In brief, PCR analysis of genomic DNA was performed using the primers (5′-3’) TAATCTGTCCTCACTCTGCC, CAGTTGACATACCTTAAT and TGCATTAATGTGCAGTGT. QuickExtract^TM^ DNA Extraction Solution (Epicentre Biotechnologies, Madison, WI) was used to isolate the genomic DNA, and MyTaq^TM^ DNA Polymerase was used to perform the PCR. PCR products were analyzed on a 2% agarose gel. Product sizes were 266 bp (wild-type) and 363 bp (homozygous reeler). The animals were humanely handled and sacrificed via cervical dislocation in accordance with the guidelines of the European Union Council (Directive 2010/63/UE) and Spanish Royal Decree (BOE 34/11370, 2013) concerning the protection of experimental animals.

### 2.3. Experimental Mouse Models

**Figure 1:**
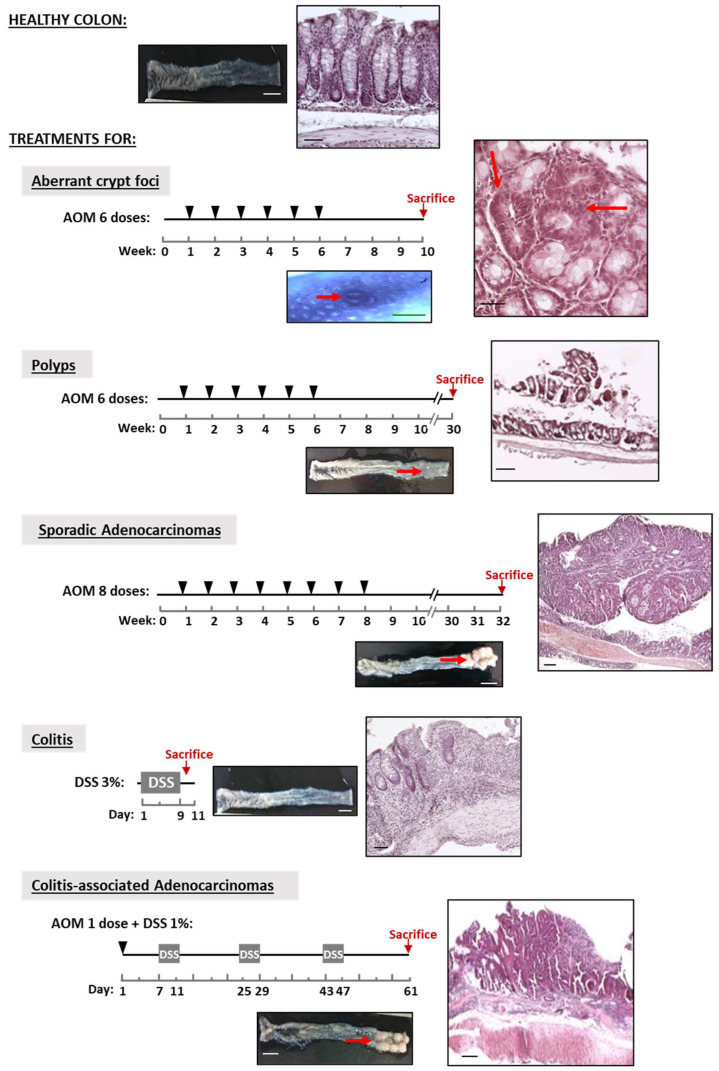
Mouse model of colon cancer progression. Experimental design of the treatments to induce lesions in the colon. Dextran sulfate sodium (DSS) was administered in the drinking water (3%) for 9 days to induce acute colitis. Azoxymethane (AOM) dissolved in PBS was administered by intraperitoneal injection at the point indicated by the arrowheads. DSS was administered in the drinking water (1%) in 4-day periods. Control groups (untreated mice) received PBS intraperitoneally and water orally. Representative photographs of mice colonic mucosa along colon cancer initiation and progression. Macroscopic (fresh tissue or stained with methylene blue solution in the case of aberrant crypt foci) and microscopic images (eosin–hematoxylin-stained sections) of mice colon with colitis, aberrant crypt foci, polyps, sporadic adenocarcinomas, colitis-associated adenocarcinomas and healthy colons (control groups). The lesions are indicated by red arrows. For each mouse model, the number of animals per experimental group was ten. White scale bars represent 1 cm, the green scale bar represents 50 µm and black scale bars represent 200 µm.

The carcinogenic agent azoxymethane (AOM, Sigma-Aldrich, Darmstadt, Germany). induces precancerous and cancerous lesions similar to those of sporadic colon cancer [24]; dextran sulfate sodium (DSS, 40 kDa, TdB Consultancy) induces inflammation, and together with AOM, produces colitis-associated adenocarcinomas [25]. Colonic lesions were induced in mice according to the experimental design shown in Figure 1. Aberrant crypt foci (ACF), polyps and adenocarcinomas were induced in 2-month-old C57BL6/J mice via the intraperitoneal injection of 10 mg AOM/kg body weight dissolved in PBS: mice received 6 weekly doses followed by an interval of either 4 weeks without treatment before the sacrifice for ACF induction or 24 weeks for polyps. Sporadic adenocarcinomas were induced with 8 weekly AOM doses and a period of 24 weeks without treatment [26]. Colitis-associated adenocarcinoma was induced in 3-month-old C57BL6/J mice following the protocol of [27]: Briefly, 10 mg AOM/kg body weight was first administered via a single intraperitoneal injection followed by 3 cycles in a 4-day period with 1% (wt/vol) DSS in drinking water. Between DSS periods, the animals drank normal water for 14 days. Acute colitis was induced in 3-month-old B6C3Fe wild-type and reeler mice by administering 3% (wt/vol) DSS in the drinking water for 9 days. Control group (untreated mice) received normal drinking water. The progression of the colon inflammation of the mice receiving DSS treatment was assessed by determining the disease activity index (DAI), histological score and mRNA levels of the pro-inflammatory cytokines, interleukin-1β (IL-1β) and tumor necrosis factor-α (TNF-α), as described previously [19]. For DAI evaluation, throughout the DSS treatment, animals were monitored daily for weight loss, stool consistency and blood in the feces (0–3 scale). Histological score (0–3 scale) was based on destruction of epithelium, dilatation of crypts, loss of goblet cells, inflammatory cell infiltrate and edema.

Control groups (untreated mice) received injections of phosphate buffer solution (PBS; in mM, 137 NaCl, 2.7 KCl, 10 Na_2_HPO_4_ and 1.8 KH_2_PO_4_; pH7.4) and normal drinking water.

### 2.4. Preparation and Evaluation of Colon Lesions in the Mouse Models

Following sacrifice, mouse colon was removed and washed with ice-cold saline solution and its length was measured, weighed and examined for the presence of tumor lesions. ACF, polyps, sporadic adenocarcinomas, colitis-associated adenocarcinomas and tissue adjacent to colon lesions were obtained via dissection with the help of a scalpel under a magnifying glass. To count the polyps and adenocarcinomas and measure their diameters, the colon was opened longitudinally, laid flat lumen side up and the entire colon was analyzed under a magnifying glass. The number of ACF was measured by using methylene blue staining and visualization under light microscope [28]. Briefly, longitudinally opened colon samples were fixed with 4% paraformaldehyde and conserved in 70% ethanol until used. For staining, colons were submerged in a freshly made 0.05% methylene blue solution, firstly in 20 dips of 1 s and then by incubation for 3 min at room temperature. After rinsing with distilled water, the ACF were counted under a light microscope with a magnification of 50× along the whole colon. All the results are expressed as the number of lesions per mouse. For histological analysis, colons were fixed by overnight incubation with PBS containing 4% paraformaldehyde and processed for eosin–hematoxylin procedure. Images captured were analyzed with the Spot Advance 3.5.4.1. program (Diagnostic Instrument, Inc.). All the assessments were carried out by two different researchers, and the result was the mean of both measurements.

### 2.5. Relative Quantification of Real-Time PCR

Total RNA was extracted from mice and human colon samples. The mouse samples (ACF, polyps, both types of adenocarcinomas, healthy colon and tissue adjacent to colon lesions) obtained by dissection were frozen in liquid nitrogen and stored at −80 °C until used. The RNeasy^®^ kit (Qiagen, Hilden, Germany) was used following the manufacturer protocol. Paraffin sections of human colon (ulcerative colitis, polyps, adenomas, adenocarcinomas and healthy) and FFPE RNeasy^®^ kit (Qiagen, Hilden, Germany) were used. Some modifications were included in the manufacturer protocol to improve the quantity and quality of the resultant RNA, such as incubation of the paraffin sections in xylene for 15 min at room temperature before starting the protocol and during it, and the washes with xylene and ethanol were repeated twice. RNA purity was assessed using spectrophotometry measurements of OD260/280 and its integrity was measured via visual inspection after electrophoresis on an agarose gel in the presence of RedSafe^TM^ (Intron Biotechnology, Seongnam-Si, South Korea) nucleic acid staining.

Once RNA was obtained, cDNA was synthesized from 1 µg of total RNA using QuantiTect^®^ reverse transcription kit (Qiagen) as described by the manufacturer. The primers used are provided in Table 1. Real-time PCR was performed with 10 µL SsoFast™ EvaGreen^®^ Supermix (BioRad, Madrid, Spain), 0.4 µM primers and 1 µL cDNA. Controls were carried out without cDNA. Amplification was run in a MiniOpticon™ System (BioRad) thermal cycler (95 °C/3 min; 35 cycles of 94 °C/40 s, 58 °C/40 s and 72 °C/40 s; 72 °C/2 min). Following amplification, a melting curve analysis was performed by heating the reactions from 65 to 95 °C in 1 °C intervals while monitoring fluorescence. Analysis confirmed a single PCR product at the predicted melting temperature. The PCR primers’ efficiencies ranged from 90 to 110%. β-actin served as the reference gene and was used for sample normalization. The cycle in which each sample crossed a fluorescence threshold, Ct, was determined. Each cDNA was run in triplicate, and the three values were averaged. Analyses of PCR were performed using the comparative Ct method with the Gene Expression Macro software supplied by BioRad.

### 2.6. Western Blot Assays

SDS-PAGE was performed on a 10% polyacrilamide gel. The lysis buffer contained: 150 mM NaCl, 1% NP-40, 0.5% sodium deoxycholate, 0.1% of sodium dodecyl sulfate, 1 mM phenylmethylsulfonyl fluoride, 20 µg/mL aprotinin, 10 µg/mL leupeptin and 50 mM Tris-HCl, pH 8. The reagents were obtained from Sigma-Aldrich, Spain. Protein was extracted, as described in [19], from distal colon segments (frozen in liquid nitrogen and stored at −80 °C) of B6C3Fe wild-type and reeler (rl−/rl−) 3-month-old mice either untreated (control) or treated with DSS for 9 days to induce acute colitis. Briefly, the tissue samples were homogenized in lysis buffer using a polytron homogenizer and incubated at 4 °C for 10 min on a rotating shaker, followed by centrifugation at 14,000× *g* for 30 min. The resultant supernatant was dissolved in the Laemmli sample buffer. A total of 20 μg of protein was loaded to each lane, electrophoresed and electrotransferred onto a nitrocellulose membrane, and the immunoreactive bands were viewed using a chemiluminescence procedure (GE Healthcare Select^®^) (Figures S1 and S2). Anti-β-actin antibody was used to normalize band density values. The relative abundance of the bands was quantified using the Image J program version 1.46 (National Institutes for Health, http://rsb.info.nih.gov/ij/index.html accessed on 18 September 2022, Bethesda, MD, USA). Protein was measured with the Bradford method [29] using gamma globulin as the standard. The antibodies and the dilutions used were anti-p53 (sc-6243) 1:500, anti-GSK-3β (sc-9166) 1:500 and anti-Akt phosphorylated (Ser^473^) (sc-7985-R) 1:500 from Santa Cruz Biotechnology; anti-p53 phosphorylated (Ser^15^) (AF1043) 1:500 from RD systems; and anti-β-actin (A2547) 1:5000 from Sigma-Aldrich, Spain. Biotinylated peroxidase-conjugated anti-mouse IgG (BA-9200) and anti-rabbit IgG (BA-1000) were obtained from Vector 1:8000.

### 2.7. Statistical Analysis

Data are presented as mean ± SEM. The number of biological samples (*n*) is equal to the number of animals or subjects used for each condition and is indicated in the figure legends. One-way ANOVA followed by Tukey´s test was used (GraphPad Prism Program v8.0). Differences were set to be significant for *p* < 0.05.

## 3. Results

### 3.1. Human and Mouse Models for Studying Colon Cancer Initiation and Progression

We started the work by analyzing samples from healthy (control) and pathological human colons and from the colons of two mouse models that mimic the progression toward either human sporadic colon adenocarcinoma or colitis-associated adenocarcinoma (see Methods, Section 2.4).

**Figure 2:**
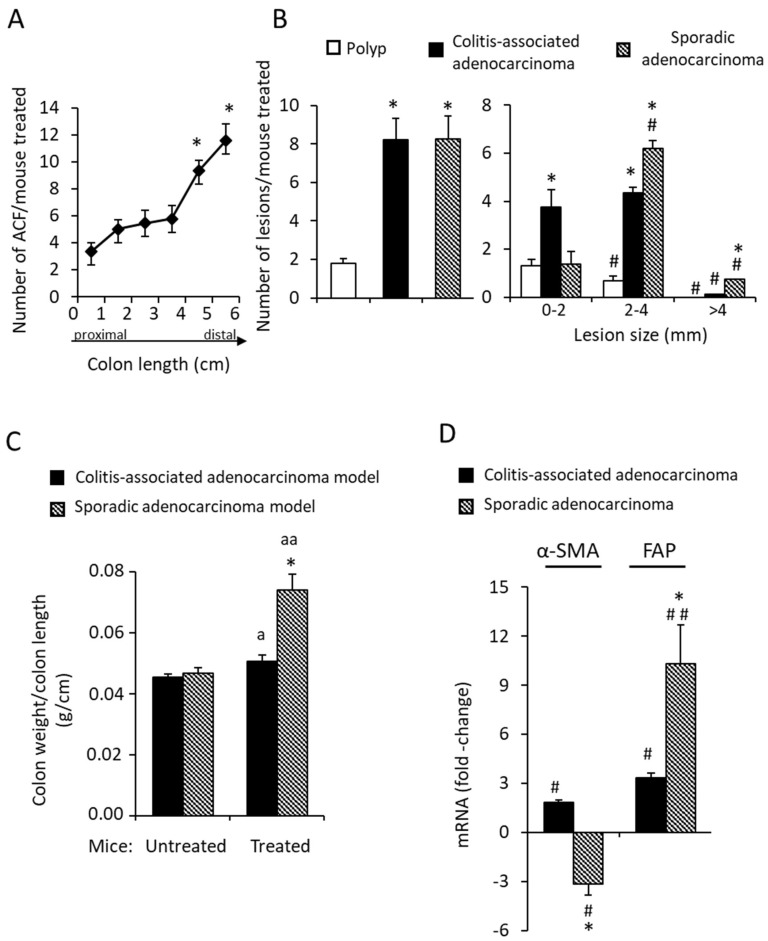
Characterization of colon lesions in the mouse models. (**A**) Number of aberrant crypt foci (ACF) per mouse observed along the colon: 0–2 cm corresponds to proximal colon, 3–4 cm to proximal–distal colon region and 5–6 cm to distal colon. (**B**) Number of lesions per mouse in the different models and sorted by size. (**C**) Ratio of colon weight/colon length expressed in g/cm. (**D**) α-SMA and FAP mRNA abundance in colon sporadic adenocarcinomas and colitis-associated adenocarcinomas expressed as fold change relative to control (untreated) mouse colons. Data are means ± SEM (*n* = 8–10 animals per group). ANOVA shows the effect (*p* < 0.0001) of the colon region on the number of ACF (in (**A**)); the type of pathology on the number and size of lesions (in (**B**)); and the type of adenocarcinoma on the ratio colon weight/colon length (in (**C**)) and the α-SMA and FAP mRNA abundances (in (**D**)). Tukey’s test: * *p* < 0.001 vs. 1 centimeter (in (**A**)); * *p* < 0.001 vs. polyps; # *p* < 0.001 vs. 0–2 mm lesions (in (**B**)); ^a^ *p* < 0.05, ^aa^ *p* < 0.001 vs. control mice and * *p* < 0.001 vs. colitis-associated adenocarcinomas (in (**C**)); # *p* < 0.05, ## *p* < 0.001 vs control mice and * *p* < 0.001 vs. colitis-associated adenocarcinomas (in (**D**)).

Representative images of the colons’ macroscopic appearances and histopathological analysis (Figure 1) reveal that the two mouse experimental models do generate the sequence of healthy colon-ACF-polyp-sporadic adenocarcinoma and that of healthy colon-colitis-associated adenocarcinoma. The untreated mice (control group) exhibited healthy colons with no lesions in them. The analysis reveals the typical characteristics of each lesion: (i) loss of crypts and infiltration of inflammatory cells in the colitis; (ii) ACFs with a thicker epithelium and higher pericryptal space; (iii) polyps with a superficial appearance with low/medium dysplasia grade; (iv) sporadic adenocarcinomas that are more protuberant, showing a high dysplasia grade in the mucosa; and (v) colitis-associated adenocarcinomas that are flatter and show a high dysplasia grade. The quantification of the lesions (Figure 2A,B) reveals that: (i) ACF were distributed throughout the colon, being at their maximum in the distal colon; (ii) the number of adenocarcinomas was higher than that of polyps; (iii) the number of both types of adenocarcinomas per mouse was practically similar; (iv) the polyps were the smallest, and sporadic adenocarcinomas were the biggest lesions in size; and (v) the ratio of colon weight/length was higher in the sporadic than in the colitis-associated adenocarcinomas (Figure 2C), indicative of greater tumoral mass.

Two cancer-associated fibroblast markers—the alpha-smooth muscle actin (α-SMA) and fibroblast activation protein alpha (FAP)—were also determined in the two adenocarcinomas. Figure 2D reveals that, relative to control mice, α-SMA mRNA abundance increases in the colitis-associated adenocarcinoma and decreases in the sporadic. The FAP mRNA levels increased in both types of adenocarcinomas, with a significantly higher increase in the sporadic adenocarcinoma. Since decreased α-SMA expression and increased FAP are associated with the most aggressive cell phenotype [30], the current observations suggest that sporadic adenocarcinoma is more aggressive than the one associated with colitis.

**Figure 3:**
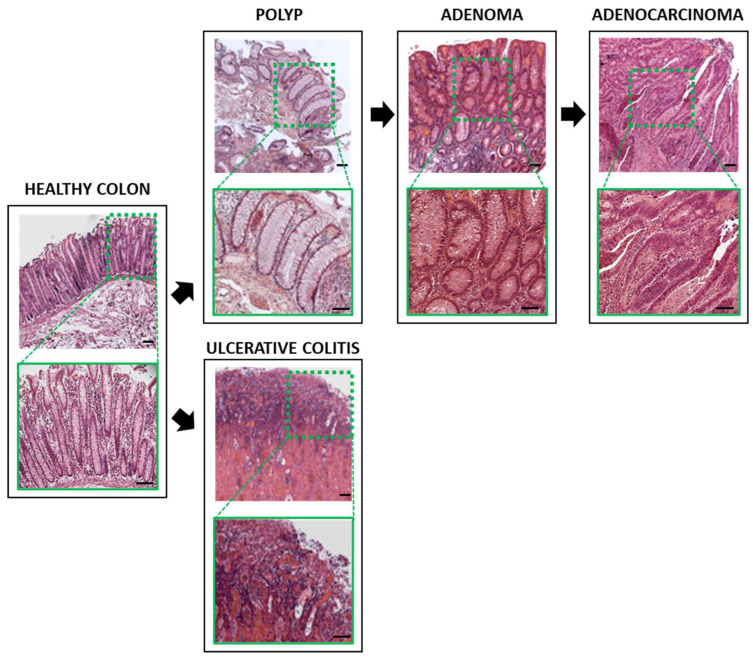
Representative photographs of healthy and pathological human colons. Eosin–hematoxylin-stained, embedded paraffin sections (5–10 µm) of healthy colon, ulcerative colitis, polyps, adenomas and adenocarcinomas are shown. The number of subjects used for each condition was eight. Scale bars represent 100 µm.

Figure 3 shows representative images of human healthy colons (control), colons from patients with ulcerative colitis and lesions developed during colon cancer progression: Polyp, adenoma and adenocarcinoma. All the samples show the typical histological alterations corresponding to the ulcerative colitis and the adenoma–carcinoma sequences.

### 3.2. Reelin, DNMT-1 and ApoER2 mRNA Abundance in Human and Mouse Colon Cancer Development

Since we previously reported, reelin downregulation in human colon adenocarcinoma together with upregulation of both DNMT-1 and ApoER2 [21], we next examined how these genes behave in each type of colon lesion. For this purpose, the relative abundances of their mRNA were measured with real-time PCR in both healthy and pathological human colons and in colon samples from the two mouse models.

**Figure 4:**
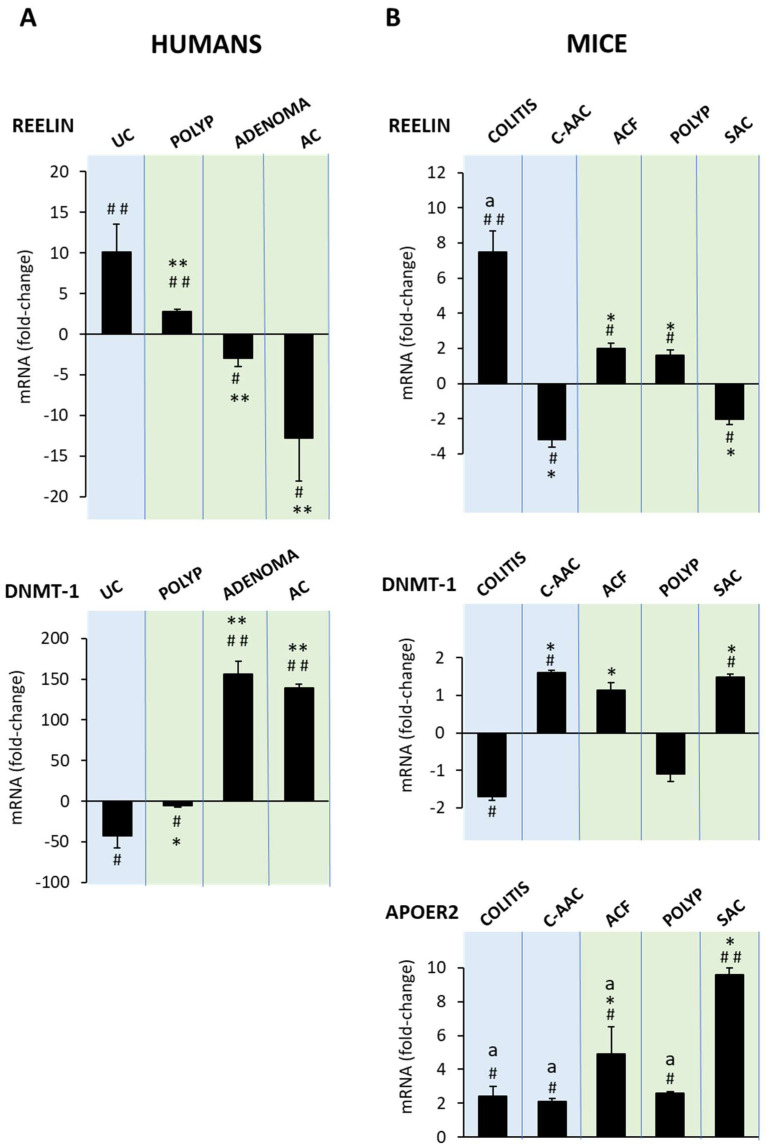
Reelin, DNMT-1 and ApoER2 mRNA abundance in human and mouse colon cancer development. Histograms represent mRNA abundance expressed as fold changes relative to healthy colons. Data are means ± SEM (*n* = 8 human or animal samples per condition). ANOVA shows an effect (*p* < 0.0001) of colonic disease progression on mRNA reelin, DNMT-1 and ApoER2 abundances. (**A**) Human tissue samples: ulcerative colitis (UC), polyp, adenoma, adenocarcinoma (AC). Tukey’s test: # *p* < 0.05; ## *p* < 0.01 vs. healthy colon and * *p* < 0.01; ** *p* < 0.001 vs. ulcerative colitis. (**B**) Mouse tissue samples: colitis, colitis-associated adenocarcinoma (C-AAC), aberrant crypt foci (ACF), polyp, sporadic adenocarcinoma (SAC). Tukey’s test: # *p* < 0.05; ## *p* < 0.01 vs. healthy colon and * *p* < 0.001 vs colitis; ^a^ *p* < 0.001 vs. sporadic adenocarcinoma.

Figure 4A shows that in humans, the lesion vs.healthy colon reelin mRNA expression (as a fold change) increases in ulcerative colitis and, to a lesser extent, in polyps and decreases in adenomas and even more in adenocarcinomas. That is, reelin mRNA abundance decreases as disease severity progresses from ulcerative colitis to adenocarcinoma, and the shift from reelin upregulation to repression occurs in the progression from non-adenomatous polyps to adenoma. The results obtained in mice (Figure 4B) were similar to those in humans: reelin mRNA expression significantly increases in colitis and in colon precancerous lesions (ACF and polyps), and it decreases in adenocarcinomas induced by either AOM or AOM-DSS treatments. As in human colons, the shift from reelin upregulation to repression occurs in the progression from polyp to the tumoral lesion.

Figure 4A also shows that, compared with a healthy colon, during the progression from colitis or precancerous lesions to colon cancer, human DNMT-1 mRNA abundance changes in the opposite direction to that of reelin. In mice (Figure 4B), DNMT-1 mRNA changes are smaller than in humans, although those measured in ACF and polyps were not significant.

ApoER2 mRNA abundance increases in all the lesions studied (Figure 4B), making it difficult to evaluate the ApoER2 contribution to the process under study. However, as compared with all the other lesions, sporadic adenocarcinoma has the highest ApoER2 mRNA expression, suggesting that, in this adenocarcinoma, ApoER2 acts as a negative regulator of reelin, as previously shown in neuroblastoma cells [22].

Altogether, the results described so far indicate that in both, humans and mice, mRNA reelin expression changes from upregulation under inflammatory conditions and precancerous lesions to repression in adenoma and adenocarcinomas. In humans, these changes could be, at least in part, mediated by DNMT-1; in mice colons, the DNMT-1 contribution appears to be less relevant. ApoER2 involvement in colonic pathology progression toward cancer is not evident from the current observations. The data also reveal that the mouse models exhibited the same phenocopy features as the human colon disease and validate their use in studying the molecular mechanisms of human cancer development.

### 3.3. Reelin, DNMT-1 and ApoER2 mRNA Expression in Tissues Adjacent to Precancerous and Cancerous Lesions in Mouse Colons

Up to now, the data obtained from the damaged colons were compared with those from the colons of healthy animals. We considered of interest to find out whether the tissue surrounding (adjacent to) the colon polyp and adenocarcinoma exhibits changes in reelin, DNMT-1 and ApoER2 mRNA expression.

**Figure 5:**
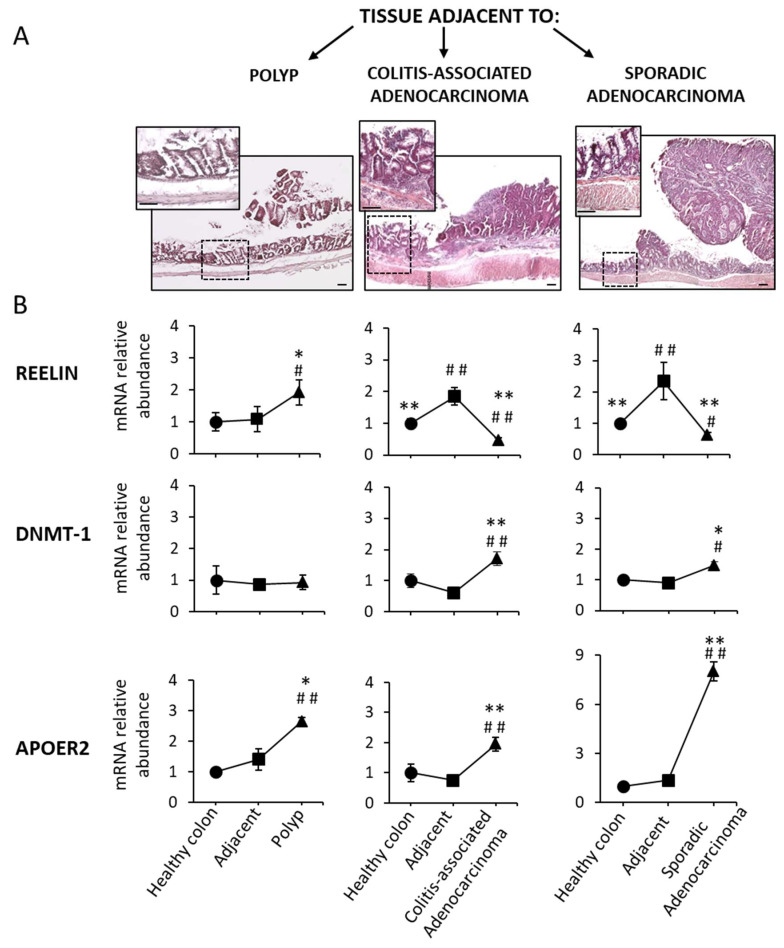
Reelin, DNMT-1 and ApoER2 mRNA relative abundance in the tissue adjacent to polyps and adenocarcinomas in mouse colons. (**A**) Representative photographs of each lesion and its adjacent tissue (inserts). In total, 5 µm embedded paraffin sections were stained with an eosin–hematoxylin procedure. Scale bars represent 200 µm. (**B**) Graphs represent mRNA abundance of reelin, DNMT-1 and ApoER2 in the healthy colon (control), in the tissue adjacent to each lesion and in the lesion itself (either polyp, colitis-associated adenocarcinoma or sporadic adenocarcinoma). The mRNA abundance value obtained for each gene was calculated considering that of the healthy colon value 1. Data are means ± SEM (*n* = 10 animals per group). ANOVA shows significant differences (*p* < 0.01) in reelin, DNMT-1 and ApoER2 mRNA abundance between adjacent tissues and the lesions. Tukey’s test: # *p* < 0.05, ## *p* < 0.001 vs. healthy colon; * *p* < 0.05, ** *p* < 0.001 vs. adjacent tissue.

Figure 5A shows representative eosin–hematoxylin-stained sections of mouse colon containing polyps, colitis-associated adenocarcinomas or sporadic adenocarcinomas, together with the amplified adjacent area of each lesion. Figure 5B summarizes the mRNA abundance of reelin, DNMT-1 and ApoER2 measured in the area close to the lesion, in the lesion itself and in healthy colons obtained from control mice. Reelin mRNA expression in the areas adjacent to the polyps was similar to that of healthy colons but significantly lower than in the polyps themselves. Regarding adenocarcinomas, reelin mRNA levels were higher in the adjacent tissues than in either healthy colons or adenocarcinomas. DNMT-1 expression in the tissue adjacent to any of the three types of lesions did not significantly differ from that in a healthy colon, nor in the polyps, but it was lower than in the adenocarcinomas. ApoER2 expression levels in the adjacent tissues were similar to the healthy colon, lower than in either polyps or the colitis-associated adenocarcinomas and much lower than in sporadic adenocarcinoma. Therefore, each gene is differently regulated in the tissue adjacent to the lesion than in its respective lesion.

Based in these observations, it can be suggested that reelin mRNA increases in the adjacent areas in response to the damage that might progress to cancer, but this change does not occur as long as reelin is present. DNMT-1 and ApoER2 might downregulate reelin expression in both types of adenocarcinomas.

### 3.4. Reelin and p53 Expression and Activation in Mouse Colon

**Figure 6:**
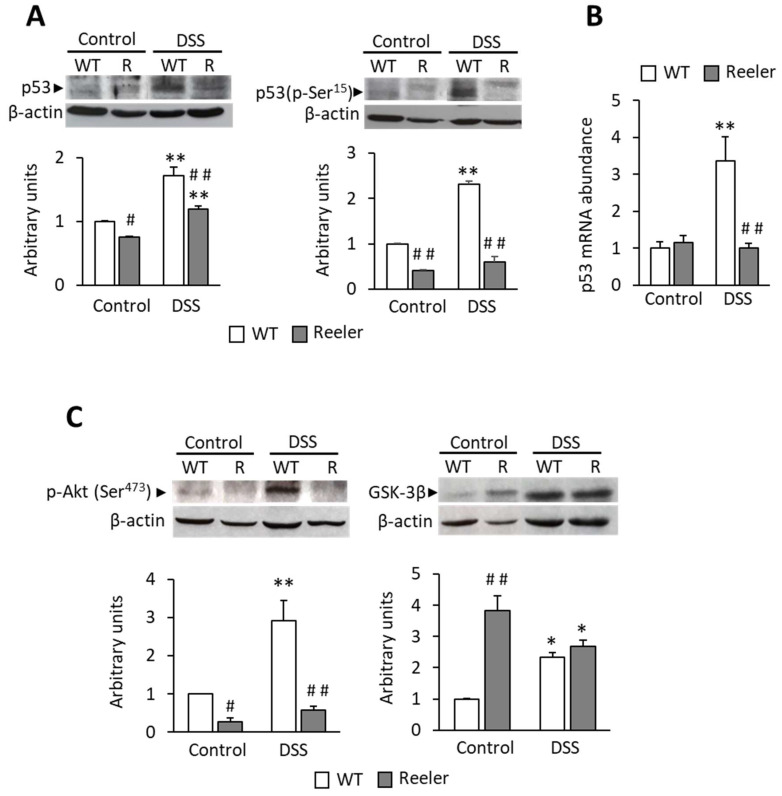
p53, phospho-Ser^15^ p53, phospho-Ser^473^ Akt and GSK-3β expression in wild-type and reeler mouse colons. Distal colon of 3-month-old wild-type (WT) and reeler (R) mice either untreated (control) or treated for 9 days with DSS were used for Western blot (**A**,**C**) or for real-time PCR assays (**B**). (**A**) Total p53 and activated p53 (p-Ser^15^) protein expressions. (**B**) p53 mRNA relative abundance. (**C**) p-Akt (Ser^473^) and GSK-3β protein expressions. Histograms represent protein or mRNA relative quantification in arbitrary units as means ± SEM (*n* = 3–5 animals per group). The protein or mRNA value measured in WT untreated mice was set to 1. ANOVA shows the effect (*p* < 0.0001) of DSS treatment and reeler mutation on protein and mRNA abundance. Tukey’s test: * *p* < 0.05, ** *p* < 0.001 vs control; # *p* < 0.05, ## *p* < 0.001 vs. WT.

To find out the mechanism by which reelin protects the colon from pathology, we examined the effect of reelin on p53 expression and activation in the distal colon of both wild-type and reeler (deficient in reelin) mice, which were either untreated (control group) or treated with DSS for 9 days (DSS group) to induce acute colitis. Total p53 and activated p53 (phosphorylated at Ser^15^) proteins were measured with Western blot. Under control conditions, the absence of reelin decreases both, the total and the phosphorylated p53 (Figure 6A). DSS treatment increased the total p53 protein in both types of mice and it increased the phosphorylated p53 protein only in wild-type mice. The DSS-induced increase in total p53 was higher in wild-type than in reeler mice.

We next evaluated the relative abundance of p53 mRNA to determine whether the changes in p53 protein resulted from changes in its gene transcription. Figure 6B shows that p53 mRNA abundance follows a pattern similar to that of the p53 protein, with the exception of DSS-untreated reeler mice: p53 mRNA does not decrease as the protein does. Altogether, the data indicate that reelin is required for p53 transcription and activation through phosphorylation-Ser^15^ and that acute colon inflammation increases p53 transcription and activation.

### 3.5. Reelin and Akt/GSK-3β Signaling in Mouse Colon

As Akt/GSK-3β signaling modulates p53 activation, we wondered whether reelin uses this signaling pathway to control p53 in the colon. We evaluated (Western blot assays) Akt phosphorylated at Ser^473^ (p-Akt) and non-phosphorylated GSK-3β (active form) proteins in the same mice colon samples used to measure p53 abundance. Figure 6C shows that DSS treatment increases p-Akt in wild-type mice and that reelin is required for p-Akt expression in DSS-treated and untreated mice. The absence of reelin upregulates GSK-3β protein expression in untreated mice, but it has no effect on DSS-treated mice. These results indicate that in mice colon reelin is necessary for Akt phosphorylation at Ser^473^, independently of the pathophysiological conditions, whereas reelin inhibits GSK-3β activation only under physiological conditions.

Overall, it can be suggested that, in mouse colons, reelin could activate Akt and inhibit GSK-3β, which in turn promotes p53 expression and activation.

## 4. Discussion

Colon cancer development is a sequential process that begins with the transformation of the normal colon with early pathological events, such as inflammation or precancerous lesions, and ultimately, with carcinoma. Most studies on changes in reelin expression in cancers have only looked at a particular stage of the cancer process, those measuring reelin expression during cancer progression being very few [16]. The current data suggest that the grade of colon reelin expression indicates the stage of this transition. They also suggest that initial colon injuries, such as colitis or precancerous lesions, upregulate reelin, which prevents the transformation toward cancerous lesions, whereas its repression favors colon cancer progression. Thus, in both mouse and human colons, reelin mRNA abundance increases in colitis and precancerous lesions and, thereafter, decreases as the injury severity increases throughout the cancer progression. Reelin expression reduction coincides with the shift from non-adenomatous polyps to adenoma in humans, and from polyps to adenocarcinomas in mice. In addition, reelin mRNA abundance in polyps or adenocarcinomas in mouse colons is lower and higher than in their adjacent tissues, respectively. It is worth noting that the tissue surrounding colon neoplastic lesions regulates cancer cell behavior, as its structure and composition become disorganized, allowing cancer progression [31]. The present data are in line with those showing that: (i) high reelin expression in the early stages of cancer and a correlation between low reelin expression and more advanced stages of gastric, lung, breast, glioblastoma, neuroblastoma and glioma cancers [32,33,34,35,36], though increased reelin expression was observed in higher grades of prostate cancer [37], and (ii) high reelin expression in areas adjacent to breast cancer [38] and glioblastoma [39].

Reelin expression during colon cancer progression appears to be, at least “in part”, regulated by the DNMT-1-mediated methylation of its promotor region. Thus, reelin and DNMT-1 mRNA levels change in opposite directions throughout colon cancer progression in both humans and mice, and the shift in the expression of both genes occurs at the same stage. Reduced reelin expression is associated with increased DNMT-1 in gastric and breast cancers [32,40], and a gradual increase in DNMT-1 mRNA abundance from ulcerative colitis to colitis-associated colorectal tumors has been reported [41,42].

We previously reported that ApoER2 appears to negatively regulate reelin expression in human colon adenocarcinoma [21]. Our data rule out such a role during colon cancer progression: ApoER2 mRNA abundance in polyps is similar to that of colitis-associated adenocarcinomas, even though their reelin expression changes in opposite directions. However, as reported for breast cancer [43], ApoER2 may contribute to cellular aggressiveness in colon adenocarcinomas. Thus, its mRNA levels are much higher in sporadic than in colitis-associated adenocarcinoma, exhibiting, however, similar decreases in reelin mRNA abundance. Therefore, the expression levels of reelin together with those of ApoER2 could contribute to the difference between the two types of adenocarcinomas. Additional data indicate that sporadic is more aggressive than colitis-associated adenocarcinoma: the former is bigger in size, has lower α-SMA mRNA abundance and higher FAP mRNA abundance, the two latter features being indicative of a more aggressive cell phenotype [31]. These observations agree with the different molecular pathogenesis of the two types of adenocarcinoma [44].

To elucidate the mechanism by which reelin protects the colon from pathology, we looked at the expression of transcriptional factor p53, one of the most altered genes in colorectal cancer [44]. As previously reported [7,11], p53 increases in response to inflammation, which might inhibit inflammatory responses and reduce the oncogenic effects of chronic inflammation. Reelin might protect the colon from pathology by regulating p53 expression. Thus, reelin seems to be required (i) to maintain the content of total p53 and p53 (p-Ser^15^) protein under physiological conditions, ii) to maintain p53 (p-Ser^15^) in DSS-induced colitis and (iii) for p53 transcription. In addition, either the absence of reelin [19,20] or the absence of p53 [45,46] increases the susceptibility of mice to developing colitis and colitis associated-colon cancer. This is the first report connecting reelin and p53.

Akt activation and subsequent GSK-3β inactivation inhibit p53 degradation, promoting its stabilization and accumulation [10]. Here, we show that under physiological conditions the lack of reelin downregulates p53 and p-Akt proteins and increases that of GSK-3β, suggesting that reelin maintains p53 at sufficient levels through p-Akt/inactivated GSK-3β. In DSS-induced colitis, the effect of reelin on p53 appears to be independent of GSK-3β because the DSS treatment increases GSK-3β protein content, the increase being reelin-independent. In neuronal and non-neuronal tissues, one of the pathways mediating reelin effects is the activation of Akt (p-Akt), which, in turn, affects different targets, including GSK-3β inactivation [17,18].

## 5. Conclusions

This is the first report showing that reelin mRNA abundance depends on the severity of the colon pathology, suggesting that reelin upregulation in response to initial injuries might prevent the beginning of colon cancer, whereas reelin repression favors it. The changes in reelin expression could be, at least in part, mediated by DNMT-1. One of the mechanisms by which reelin protects the colon from pathology could be increased p53 transcription and protein activation via p-Akt and GSK-3β inactivation. We propose reelin as a biomarker to predict colon pathology progression since specific, relative levels of its expression could be established at each stage of pathology progression.

## 6. Patents

“Use of reelin as a biomarker of intestinal diseases”. Appl. No. 201930195. Date of presentation: 4 March 2019. Assignee: Universidad de Sevilla, Spain. Status: Filed.

## Figures and Tables

**Table 1:** Oligonucleotide sequences used for real-time PCR assays.

Human			
Gene	GeneBank ID	Sense (5′…3′)	Antisense (5′…3′)
Reelin	NM_005045	CCACGAGAACTGATTACCAC	ATTGTGCTGACATTGGAAGG
DNMT-1	NM_001130823	CAAGTTCTGCCTATCTTGTATCC	TGATGTTGAAAGTAAAGGCCTC
**Mouse**			
Reelin	NM_011261.2	GGACTAAGAATGCTTATTTCC	GGAAGTAGAATTCATCCATCAG
DNMT-1	NM_ 010066.4	CAAGTTCTGCCTATCTTGTATCC	TGATGTTGAAAGTAAAGGCCTC
ApoER2	NM_001080926.1	GAATGAAGGCAGCCAGAT	GTTGTCGAAATTCATGCTC
α-SMA	NM_007392.3	CTTTGCTGGTGATGATGCTC	GCGAAGCTCGTTATAGAAGG
FAP	NM_007986.3	CGGGAAGCAACTCATGTCCT	TGATTCTCACTGCACAGCGT
p53	AB020317.1	CAGAAGATATCCTGCCATCACC	GGAGAGTACGTGCACATAACAG
**Human–Mouse**			
β-actin	NM_007393.3	ACCCACACTGTGCCCATCTA	CGGAACCGCTCATTGCC

## Data Availability

The datasets used and/or analyzed during the current study are available from the corresponding author upon reasonable request.

## Supplementary Materials

The following supporting information can be downloaded at: https://www.mdpi.com/article/10.3390/biology11101406/s1, Figure S1: Western blots of (A) phosphor-Ser^15^ p53 and (B) total p53 protein detected with either anti-p53 (sc-6243) 1:500 or anti-p53 phosphorylated (Ser^15^) (AF1043) 1:500, respectively. β-actin protein was detected using anti β-actin (A2547) 1:5000; Figure S2: Western blots of (A) phosphor-Ser^473^ Akt and (B) GSK 3-β protein detected with either anti-AKT phosphorylated (Ser^473^) (sc-7985-R) 1:500 or anti-GSK 3-β (sc-9166) 1:500, respectively. Β-actin protein was detected using anti β-actin (A2547) 1:5000.
