# Supplementary material for: Reelin Protects against Colon Pathology via p53 and May Be a Biomarker for Colon Cancer Progression
Source: Biology (Basel). 2022 Sep 26;11(10):1406. doi: 10.3390/biology11101406 (PMC9598338; doi:10.3390/biology11101406)
Supplement: Supplementary file 1 [file biology-11-01406-s001.zip › biology-1881099-supplementary.pdf]

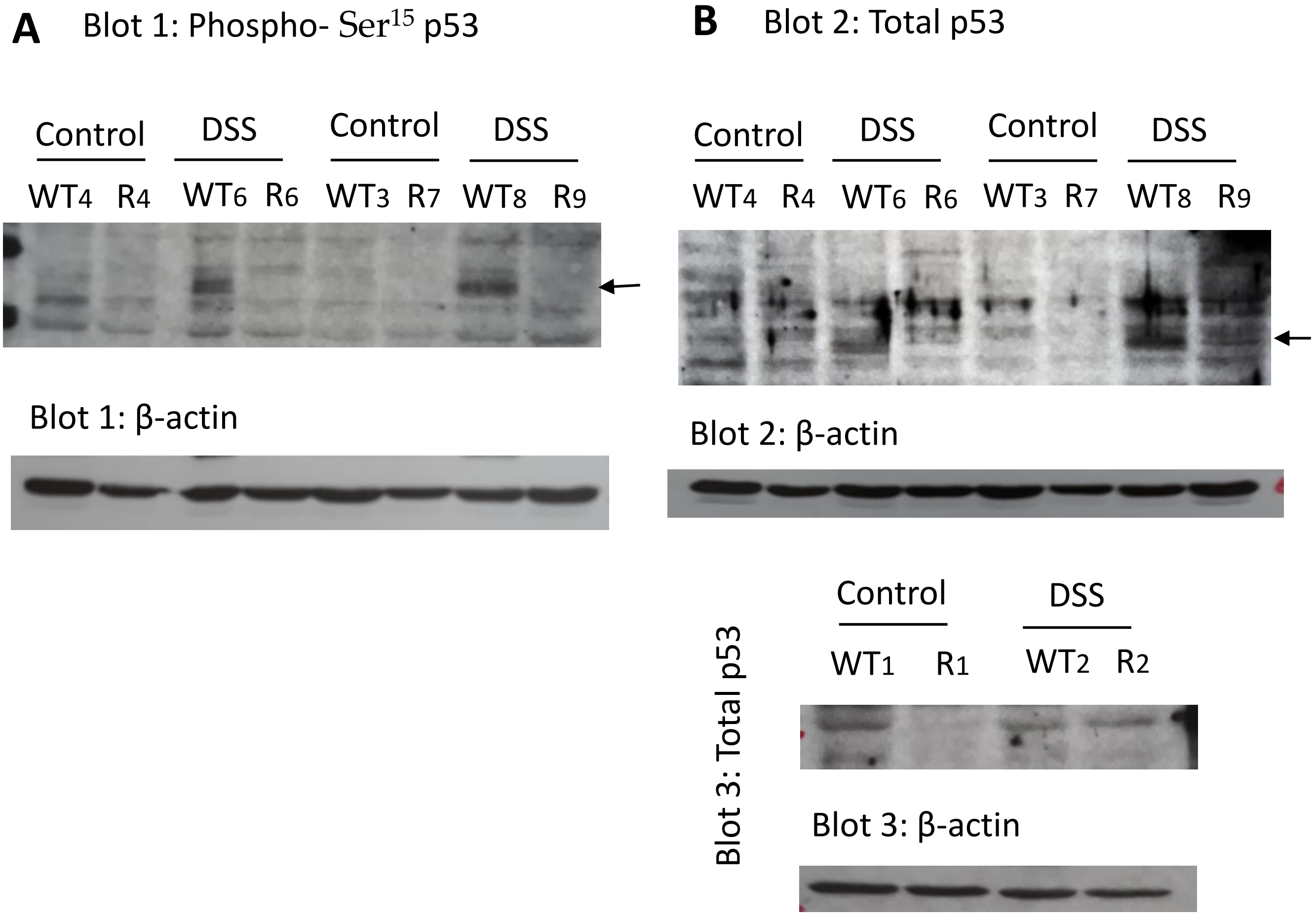

Figure S1: Western blots of (A) phospho- Ser^{15} p53 and (B) total p53 protein detected with either anti-p53 (sc-6243) 1:500 or anti-p53 phosphorylated (Ser^{15}) (AF1043) 1:500, respectively. β-actin protein was detected using anti β-actin (A2547) 1:5,000.

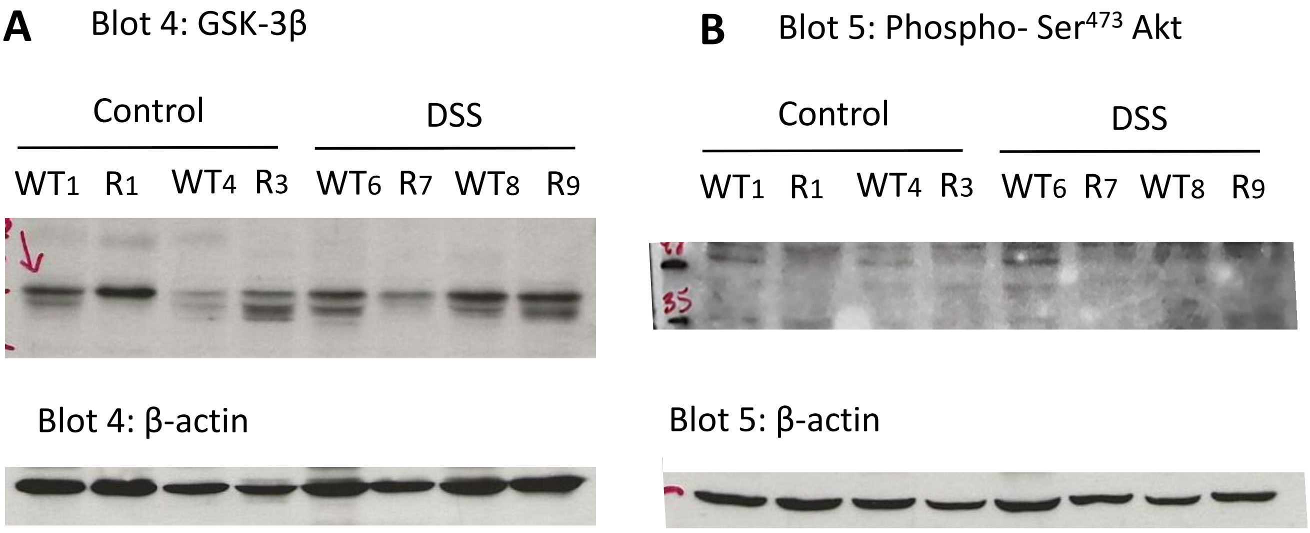

Figure S2: Western blots of (A) phospho- Ser^{473} Akt and (B) GSK-3β protein detected with either anti-AKT phosphorylated (Ser^{473}) (sc-7985-R) 1:500 or anti-GSK-3β (sc-9166) 1:500, respectively. β-actin protein was detected using anti β-actin (A2547) 1:5,000.
